# Distinct medication-state modulation of motor-cortical low-beta power in tremor-dominant and postural instability/gait difficulty Parkinson’s disease: a source-space resting-state EEG study

**DOI:** 10.3389/fneur.2026.1841473

**Published:** 2026-06-25

**Authors:** Hao Wang, Xiangyu Chen, Guoqing Wu, Xiaowei Du, Ganqin Du

**Affiliations:** 1 The First Affiliated Hospital of Henan University of Science and Technology, Luoyang, Henan, China; 2 Clinical Medical College, Henan University of Science and Technology, Luoyang, Henan, China

**Keywords:** beta oscillations, motor cortex, Parkinson’s disease, postural instability/gait difficulty, source-space EEG, tremor-dominant

## Abstract

**Background:**

Source-space electroencephalography (EEG) provides a noninvasive approach for probing oscillatory activity in motor-cortical and cerebellar networks in Parkinson’s disease (PD). However, electrophysiological evidence differentiating tremor-dominant (TD) and postural instability/gait difficulty (PIGD) subtypes under a harmonized medication OFF/ON protocol remains limited. We therefore examined whether TD and PIGD differ in medication-state modulation of motor-cortical low-beta power and cerebellum-related connectivity using 64-channel resting-state source-space EEG.

**Methods:**

We prospectively enrolled 12 healthy controls, 20 patients with PIGD, and 18 with TD. In a fixed OFF-to-ON sequence, participants underwent 64-channel eyes-closed resting-state EEG. Source-space power spectral density (PSD) and weighted phase lag index (wPLI) were computed for prespecified motor-cortical and cerebellar regions of interest. Primary effects were tested with linear mixed-effects models. Exploratory edge-wise analyses were controlled using the Benjamini–Hochberg false discovery rate (BH-FDR), and network-level effects were assessed with network-based statistics (NBS).

**Results:**

For the prespecified primary PSD endpoint, low-beta relative PSD in the primary motor cortex (M1) showed a significant group × state interaction (χ^2^(1) = 5.84, *p* = 0.016; *β* = −1.45 dB, 95% CI [−2.59, −0.32]). From OFF to ON, low-beta power increased in the PIGD group (+1.26 dB, 95% CI [0.48, 2.04]) but showed little change in the TD group (−0.20 dB, 95% CI [−1.02, 0.63]). This interaction remained stable in influence and leave-one-out analyses. The prespecified primary wPLI endpoint was not significant (*p* = 0.153). In exploratory analyses, high-beta (21–35 Hz) ΔwPLI between the right cerebellar Crus I and right lobule VI differed between subtypes and survived within-band edge-wise BH-FDR correction (*q* = 0.028), whereas NBS identified no significant network-level component.

**Conclusion:**

Tremor-dominant and PIGD showed distinct medication-state modulation of motor-cortical low-beta power, with M1 low-beta relative PSD emerging as the most consistent electrophysiological difference in this cohort. By contrast, cerebellum-related connectivity findings were limited to exploratory edge-wise effects and require independent replication.

## Introduction

Parkinson’s disease (PD) is a clinically heterogeneous neurodegenerative disorder with substantial variability in motor phenotype, disease progression, and treatment response (1, 2). Among the most widely used motor classifications are the tremor-dominant (TD) and postural instability/gait difficulty (PIGD) subtypes, which differ in functional burden, risk of falls and freezing, and long-term prognosis (1–7). However, TD and PIGD are clinical phenotypes rather than fully separated circuit entities, and subtype assignment based on clinical rating scales alone may be influenced by disease duration, medication exposure, and phenotypic transition over time (2, 4, 5). These limitations highlight the need for objective physiological correlates to refine motor subtype stratification while acknowledging overlap across clinical subtypes.

From a circuit perspective, TD and PIGD may involve partially distinct but interacting network mechanisms (8). Tremor-related manifestations have been linked to abnormal rhythmic activity within the cerebello-thalamo-cortical pathway and its interaction with basal ganglia circuits (9–16). At the same time, tremor can respond to dopaminergic therapy and deep brain stimulation (DBS), and beta-band activity, particularly in the low-beta range, has been associated with bradykinesia/rigidity, motor set maintenance, and dopaminergic modulation rather than being a purely akinetic-rigid or PIGD-specific marker (11–20). Source-space resting-state EEG provides a practical and noninvasive approach for estimating oscillatory activity in cortical motor regions and selected superficial cerebellar regions (21–23). In addition, phase-lag-based connectivity metrics such as the weighted phase lag index (wPLI) may reduce, but do not eliminate, the influence of spurious zero-lag coupling caused by volume conduction (24–26).

Accordingly, within a standardized medication OFF/ON framework, we applied a harmonized resting-state source-space EEG pipeline to test whether TD and PIGD differ in medication-state modulation patterns of motor-cortical low-beta activity, while also exploring whether cerebellum-related connectivity measures provide additional candidate physiological signals for future validation. Based on prior mechanistic studies (17, 19, 20), we prespecified the group × state interaction in M1 low-beta relative power spectral density (PSD) as the primary endpoint. Power measures from other regions of interest (ROIs) and wPLI metrics were analyzed as supportive or exploratory outcomes rather than as evidence for a simple dopamine-dependent PIGD versus cerebellar TD dichotomy.

## Methods

### Participants and clinical assessments

Patients with PD were consecutively enrolled from an early PD cohort registered at the Department of Neurology (Registry No. 221100210500), and age-matched healthy controls (HCs) were recruited in parallel. PD diagnoses and motor phenotyping were established by senior movement-disorder specialists according to Movement Disorder Society (MDS) clinical diagnostic criteria (27). Healthy controls were included for descriptive reference and pipeline quality control; all primary inferential analyses were restricted to PD participants and focused on comparisons between the TD and PIGD subtypes. Inclusion criteria were: idiopathic PD, age ≤75 years, Hoehn & Yahr stage ≤3, ability to complete eyes-closed resting-state EEG acquisition, and provision of written informed consent. Exclusion criteria were: drug-induced, vascular, or other neurodegenerative parkinsonism, or parkinsonism-plus syndromes; severe psychiatric conditions likely to substantially affect testing (e.g., severe anxiety/depression or other major psychiatric disorders); history of severe traumatic brain injury, cerebrovascular disease, or intracranial structural lesions; clinically diagnosed dementia or marked dementia suggested by MoCA/MMSE; and severe resting or action tremor rendering EEG signals unusable.

Motor severity was assessed using Part III of the MDS-Unified Parkinson’s Disease Rating Scale (MDS-UPDRS III). H&Y stage, disease duration, and levodopa equivalent daily dose [LEDD (28)] were also recorded. All PD participants underwent paired clinical and EEG assessments in medication OFF and ON states. The OFF state was defined as withdrawal of all dopaminergic medication for at least 12 h overnight before assessment. The ON assessment was performed later on the same day, approximately 1 h after the participant’s usual dopaminergic regimen, when symptoms had entered a relatively stable period. For drug-naïve patients (LEDD = 0 mg; *n* = 11), the ON assessment was performed approximately 1 h after an oral 250 mg levodopa challenge. Because OFF assessment required overnight withdrawal, the acquisition order was fixed as OFF followed by ON.

Motor subtypes were determined using the ratio of tremor to PIGD subscores derived from relevant MDS-UPDRS items (1, 3), and participants were classified as TD or PIGD accordingly. In the present study, TD/PIGD classification reflected the clinical phenotype at enrollment rather than long-term stable subtype assignment.

This study was performed in accordance with the principles of the Declaration of Helsinki and was approved by the Ethics Committee of the First Affiliated Hospital of Henan University of Science and Technology (Approval No. 2023–469). Written informed consent was obtained from all participants.

### EEG acquisition and preprocessing

Resting-state scalp electroencephalography (EEG) was recorded using a 64-channel system (Brain Vision Recorder; sampling rate, 500 Hz). Electrodes were positioned according to the international 10–10 system, and impedances were maintained below 5 kOhm throughout acquisition. Participants were instructed to remain awake, relaxed, and as still as possible with their eyes closed in a quiet environment. Each acquisition targeted at least 5 min, and original continuous EEG recordings were all longer than 5 min. In PD participants, two recordings were obtained on the same day, first in the medication OFF state and subsequently in the ON state. EEG preprocessing was performed in EEGLAB (v2025.0.0). Continuous data were band-pass filtered between 1 and 80 Hz, and a 50-Hz notch filter was applied to attenuate line noise. Channel quality was visually inspected, and channels with persistently poor signal quality were excluded from further analysis. Spatial interpolation was not performed before source reconstruction in order to avoid introducing interpolated spatial information into the inverse solution. Independent component analysis (ICA) was then conducted. Artifact-related components, including ocular and electromyographic components, were identified by ICLabel-assisted screening followed by manual review and were removed. The cleaned data were subsequently re-referenced to the common average reference. After preprocessing and artifact rejection, a continuous artifact-free segment was selected for each available recording; a 90-s segment was used whenever available. A revision-stage quality-control (QC) audit identified three ON-state PD recordings shorter than the target 90 s, including two PIGD recordings and one TD recording. These recordings were retained in the main analysis to preserve paired OFF/ON data in this modest cohort. Segment duration and post-preprocessing quality-control metrics are summarized in Supplementary Table S1. Exact upstream bad-channel deletion counts and removed ICA-component counts were not recoverable from saved post-preprocessing files unless explicitly stored in metadata. The primary inferential analysis was a source-space ROI analysis, not a whole-scalp 64-channel mass-univariate analysis.

### Source reconstruction and ROI time-series extraction

Source reconstruction was performed in template space using the ICBM152 MRI as the anatomical reference. A voxel-wise source space encompassing both cortical and cerebellar regions was generated. The forward model was computed using a three-shell spherical head model, and the inverse solution was estimated using standardized low-resolution brain electromagnetic tomography (sLORETA) with unconstrained source orientation. Regions of interest (ROIs) were defined *a priori* to capture motor-cortical and cerebellar nodes relevant to the study hypotheses. Cortical ROIs included bilateral primary motor cortex (M1) and supplementary motor area (SMA), whereas cerebellar ROIs included bilateral Crus I and lobule VI. ROI coordinates were selected based on prior meta-analyses of motor/premotor functional localization and cerebellar functional topography (21, 22). In Brainstorm, ROIs were implemented as spherical seeds. For the motor network, the Size parameter was set to 15 for left M1 (−37, −21, 58), right M1 (37, −21, 58), left SMA (−6, −6, 58), and right SMA (6, −6, 58). For the cerebellar network, the Size parameter was set to 10 for left Crus I (−36, −66, −30), right Crus I (36, −66, −30), left lobule VI (−20, −58, −20), and right lobule VI (20, −58, −20). Voxel-level source time series were extracted within each ROI. Principal component analysis (PCA) was then applied, and the first principal component was used as the representative ROI signal for subsequent analyses. We did not perform whole-scalp 64-channel mass-univariate screening; therefore, the source-space ROI results should not be interpreted as evidence that all other scalp channels were tested or negative.

### Spectral power and wPLI functional connectivity

Spectral power was estimated using Welch’s method with 2-s windows and 50% overlap. In accordance with the *a priori* hypothesis, beta activity was subdivided into low-beta (13–20 Hz) and high-beta (21–35 Hz). Relative power was calculated as band-specific power normalized to total power and then transformed to decibel units using 10 × log10 for statistical analysis. Functional connectivity was estimated using the weighted phase lag index (wPLI)19 to assess phase-lagged coupling while reducing, but not eliminating, volume-conduction-related zero-lag effects. ROI time series were transformed into analytic signals via the Hilbert transform. Windowed wPLI was computed using 2-s windows with 50% overlap and then averaged across windows to generate a static wPLI matrix for each participant and medication state. Trait-level connectivity was defined as the mean of OFF and ON values, whereas medication-state modulation was defined as ΔwPLI = wPLI_ON − wPLI_OFF.

### Statistical analysis

Baseline characteristics are presented as median (interquartile range) or counts, as appropriate. The Mann–Whitney U test or Fisher’s exact test were used to assess the differences between groups. The prespecified primary spectral endpoint was low-beta relative power in M1. Primary inference was performed using linear mixed-effects models (LMMs) with participant-specific random intercepts to account for repeated measurements and fixed covariates for age, sex, and disease duration. The primary effect of interest was the subtype × state interaction. Effect estimates are reported as regression coefficients (*β*) with 95% confidence intervals. For connectivity analyses, the prespecified primary endpoint was the trait mean of ipsilateral cerebellum–M1 low-beta wPLI. This endpoint and the corresponding subtype × state interaction were tested using analogous LMMs. Spectral measures from other ROIs and non-primary connectivity metrics were treated as supportive or exploratory outcomes. Exploratory edge-wise analyses were corrected using the Benjamini–Hochberg false discovery rate (BH-FDR) within each prespecified frequency band across the prespecified connections. To assess network-level effects, the network-based statistic (NBS) was additionally applied using 5,000 permutations and a component-forming threshold of *p* < 0.01. Robustness analyses for the primary spectral endpoint included influence diagnostics and leave-one-out analyses. Exploratory associations between electrophysiological measures and clinical change were evaluated using standard linear regression, with slopes, 95% confidence intervals, and *p*-values reported. Electrophysiological change metrics were defined as ON–OFF, whereas clinical change in MDS-UPDRS III was defined as OFF–ON, such that greater positive values indicated greater motor improvement. All statistical tests were two-sided. A *p*-value < 0.05 was considered statistically significant.

Segment-duration sensitivity analyses were added during revision. First, the 90-s analyses were repeated after excluding the three affected participants as complete paired subjects, removing both OFF and ON recordings to preserve within-participant pairing. Second, a uniform 60-s PSD and wPLI dataset was analyzed for all available recordings. Inferential models were restricted to PIGD and TD participants with complete OFF/ON pairs; HC and mixed or unknown subtypes were not included in PIGD-vs-TD inferential models. PSD models used participant-specific random intercepts with the formula outcome ~ group × state + age + sex + disease duration + (1|subject_id), with PIGD and OFF as reference levels. The primary wPLI trait model used outcome ~ group + age + sex + disease duration. Edge-wise wPLI sensitivity analyses were repeated with within-band BH-FDR correction where the edge family could be reconstructed.

### Generative AI-assisted code drafting

Generative AI tools were used as auxiliary aids to assist with analysis-code drafting and refinement, manuscript language editing, figure/table presentation checks, and organization of responses to reviewers. No data, statistical results, scientific interpretations, or conclusions were generated autonomously by AI. All analytical steps, code execution, result verification, manuscript revisions, and final interpretations were performed, checked, and approved by the authors. The authors take full responsibility for the accuracy and integrity of the submitted work.

## Results

### Baseline characteristics

The final analysis cohort included 20 patients with PIGD, 18 with TD, and 12 healthy controls. Baseline demographic and clinical characteristics are summarized in Table 1. Primary inferential analyses were restricted to the PD subtypes. The two PD subtype groups were broadly comparable in age, disease duration, LEDD, H&Y stage, and MDS-UPDRS III scores in both medication states, whereas sex distribution differed between groups. Accordingly, age, sex, and disease duration were retained as covariates in the prespecified inferential models.

**Table 1:** Baseline demographic and clinical characteristics of the study cohort.

Variable	HC	PIGD	TD	*p*
Demographics				
Age (years)	63.00 (55.75, 64.00)	61.50 (55.75, 68.00)	64.00 (62.25, 68.00)	0.151
Sex (male/female)	5/7	16/4	8/10	0.042
Cognition and mood				
MMSE score	–	25.50 (21.75, 28.00)	26.00 (24.25, 26.75)	0.701
MoCA score	–	23.00 (17.00, 26.00)	25.00 (19.25, 26.00)	0.411
HAMA score	–	9.50 (2.75, 12.25)	6.50 (3.25, 15.50)	0.758
HAMD score	–	9.00 (4.00, 14.25)	6.00 (3.50, 9.75)	0.310
Disease and medication				
Disease duration (years)	–	2.00 (1.00, 5.00)	4.00 (2.00, 6.00)	0.303
LEDD (mg/day)	–	337.50 (0.00, 600.00)	300.00 (50.00, 736.50)	0.953
Motor severity				
H&Y stage (OFF)	–	2.00 (1.00, 2.50)	2.00 (1.00, 2.50)	0.964
H&Y stage (ON)	–	2.00 (1.00, 2.50)	2.00 (1.00, 2.50)	0.964
MDS-UPDRS III score (OFF)	–	26.00 (19.75, 32.25)	24.00 (16.75, 42.50)	0.942
MDS-UPDRS III score (ON)	–	14.50 (10.00, 25.25)	16.50 (10.00, 26.00)	0.803
MDS-UPDRS III improvement rate (%)	–	43.00 (30.25, 55.25)	38.50 (27.75, 45.75)	0.334

Values are median (IQR) unless otherwise indicated; sex is presented as male/female counts; *p*-values compare PIGD vs. TD only.

### Prespecified primary spectral endpoint

**Figure 1:**
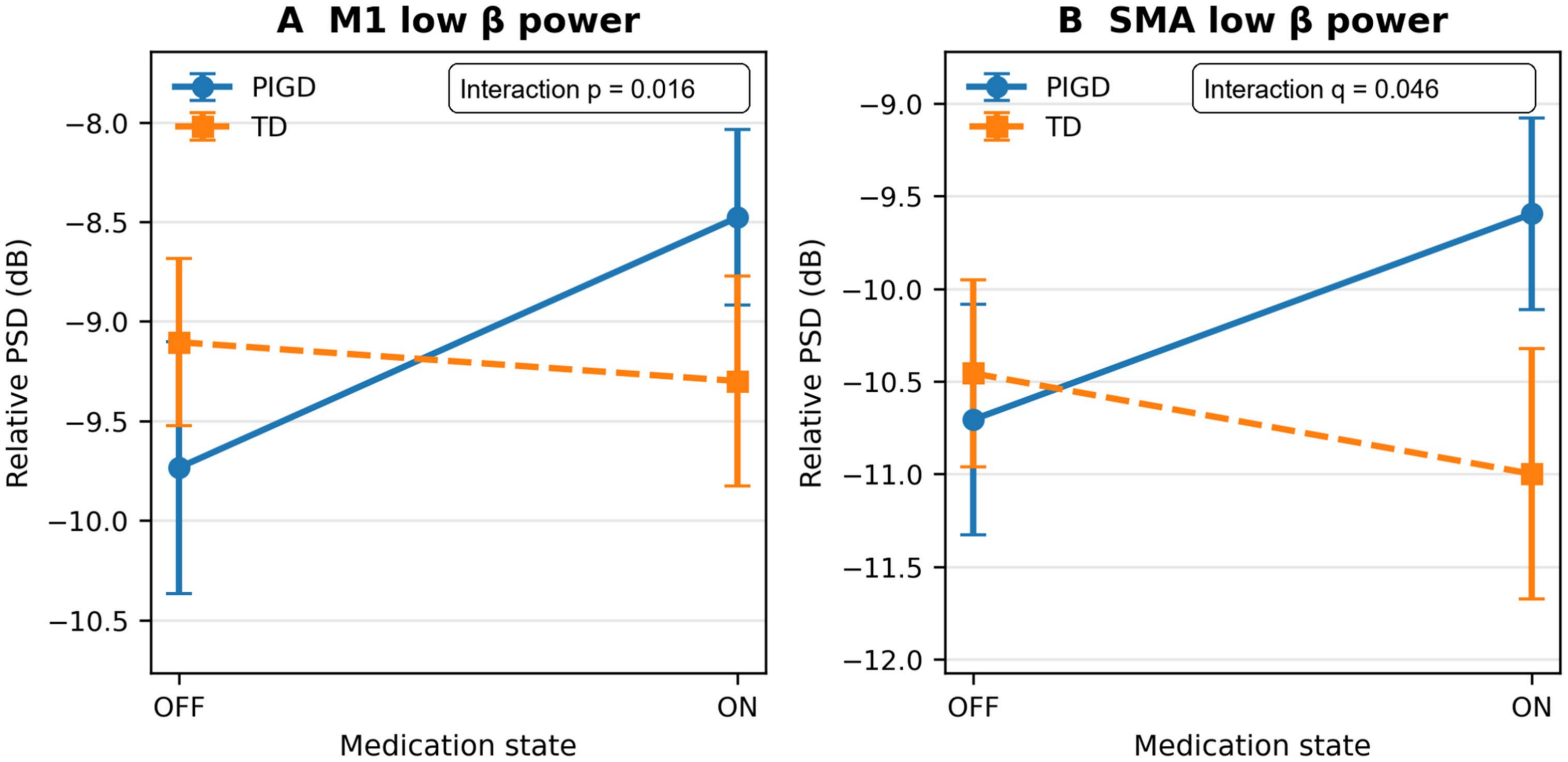
Medication-state modulation of low-beta relative power in M1 and SMA. **(A)** Low-beta relative power (13–20 Hz) in the primary motor cortex (M1) is shown by subtype and medication state. The M1 result represents the prespecified primary spectral endpoint, and the linear mixed-effects model showed a significant subtype × state interaction (*χ*^2^(1) = 5.84, *p* = 0.016). **(B)** Low-beta relative power (13–20 Hz) in the supplementary motor area (SMA) is shown by subtype and medication state. The SMA result represents a supportive endpoint and showed a subtype × state interaction after BH-FDR correction within the exploratory PSD family (*χ*^2^(1) = 6.78, *q* = 0.046).

For the prespecified primary spectral endpoint, low-beta relative power in M1 showed a significant subtype × state interaction in the linear mixed-effects model [likelihood ratio test, *χ*^2^(1) = 5.84, *p* = 0.016], as was shown in Table 2. The corresponding interaction estimate was *β* = −1.45 dB (95% CI, −2.59 to −0.32). From OFF to ON, low-beta power increased in the PIGD group (+1.26 dB, 95% CI, 0.48 to 2.04), whereas the TD group showed little change (−0.20 dB, 95% CI, −1.02 to 0.63) (Figure 1 and Table 2).

**Table 2:** Main spectral and connectivity results for prespecified, supportive, and exploratory analyses.

Domain	Endpoint	Band	Contrast	Result	Interpretation	Multiple-comparison correction
PSD	M1 relative PSD	13–20 Hz	Group × state	*χ*^2^(1) = 5.84, *p* = 0.016; *β* = −1.45 dB	Primary endpoint	Primary; no multiplicity correction
PSD	SMA relative PSD	13–20 Hz	Group × state	*χ*^2^(1) = 6.78, *q* = 0.046	Supportive endpoint	BH-FDR within PSD family
wPLI	Ipsilateral cerebellum-M1 trait wPLI	13–20 Hz	TD vs. PIGD trait	*χ*^2^(1) = 2.04, *p* = 0.153	Primary wPLI endpoint; non-significant	Primary; no multiplicity correction
wPLI	Right M1-left lobule VI trait edge	13–20 Hz	TD vs. PIGD trait	*β* = −0.0239, *q* = 0.019	Exploratory edge-wise	BH-FDR within band
wPLI	Right M1-left Crus I trait edge	13–20 Hz	TD vs. PIGD trait	*β* = −0.0185, *q* = 0.038	Exploratory edge-wise	BH-FDR within band
wPLI	Right Crus I-right lobule VI Δ edge	21–35 Hz	TD vs. PIGD Δ(ON–OFF)	*β* = 0.0407, *q* = 0.028	Exploratory edge-wise	BH-FDR within band
NBS	Network-level wPLI components	13–20/21–35 Hz	Subtype comparisons	Minimum p_FWER = 0.055	No FWER-significant component	Network-level FWER

PSD, power spectral density; wPLI, weighted phase lag index; NBS, network-based statistic; CI, confidence interval.

### Supportive spectral findings

Among non-primary spectral outcomes, low-beta relative power in SMA also showed a significant subtype × state interaction that survived correction within the exploratory PSD family [*χ*^2^(1) = 6.78, *q* = 0.046]. The direction of effect was consistent with that observed in M1, with a larger OFF-to-ON increase in the PIGD group than in the TD group (Figure 1 and Table 2). No additional spectral findings are emphasized beyond these prespecified and supportive results.

### Prespecified primary connectivity endpoint and network-level inference

For the prespecified primary connectivity endpoint, the trait mean of ipsilateral cerebellum–M1 low-beta wPLI did not differ significantly between subtypes [*χ*^2^(1) = 2.04, *p* = 0.153]. Likewise, no significant subtype × state interaction was observed for this endpoint. At the network level, NBS did not identify a significant component after family-wise error rate correction (minimum pFWER = 0.055; 5,000 permutations; component-forming threshold *p* < 0.01) (Table 2). Taken together, these results did not support a robust subtype-related difference in the prespecified cerebellum–M1 low-beta connectivity measure at either the endpoint or network level.

### Exploratory edge-wise connectivity findings

**Figure 2:**
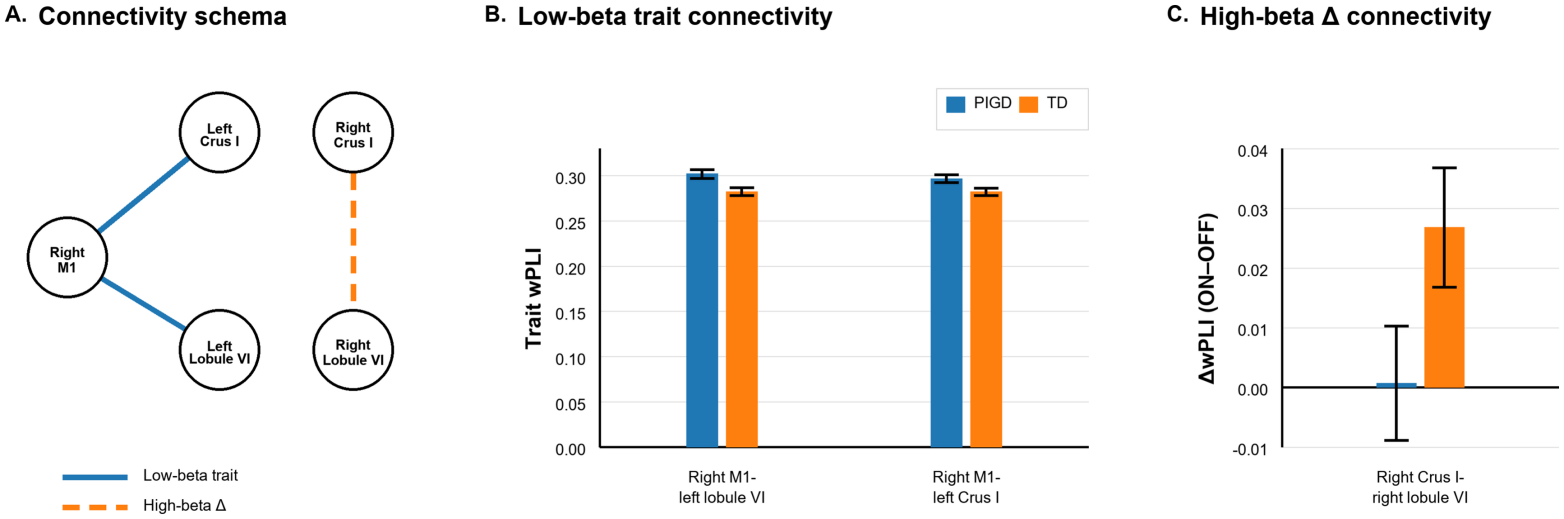
Exploratory edge-wise beta-band wPLI findings. **(A)** Connectivity schema showing the exploratory low-beta trait and high-beta ΔwPLI edges. **(B)** Low-beta trait wPLI was lower in TD than PIGD for the right M1–left lobule VI edge and right M1–left Crus I edge. **(C)** High-beta ΔwPLI was higher in TD for the right Crus I–right lobule VI edge. These findings were identified after within-band BH-FDR correction, were sparse and edge-specific, and were not supported by network-level NBS.

Exploratory edge-wise analyses identified only a small number of candidate subtype-related connectivity differences after within-band BH-FDR correction. In the low-beta range (13–20 Hz), trait wPLI was lower in the TD group than in the PIGD group for the right M1–left lobule VI edge (*β* = −0.0239, *q* = 0.019) and the right M1–left Crus I edge (*β* = −0.0185, *q* = 0.038). In the high-beta range (21–35 Hz), the TD group showed a larger OFF-to-ON increase in wPLI for the right Crus I–right lobule VI edge (*β* = 0.0407, *q* = 0.028). These effects were confined to exploratory edge-wise analyses and were not supported by network-level NBS (Figure 2 and Table 2).

### Robustness analyses for the primary spectral endpoint

Robustness analyses supported the stability of the primary M1 finding. After removal of one high-leverage observation, the subtype × state interaction for M1 low-beta power remained significant [*χ*^2^(1) = 6.11, *p* = 0.014]. In leave-one-out analyses restricted to PD participants, the interaction remained statistically significant in all 38 iterations (100% retention rate, all *p* < 0.05), indicating strong within-sample robustness of the primary spectral result (Table 3).

**Table 3:** Robustness analyses and exploratory associations with clinical improvement.

Analysis	Outcome	Result	Interpretation
Clinical OFF/ON change	MDS-UPDRS III (OFF–ON)	Median 9.0 points (IQR 4.0–13.0); *p* < 0.001	Medication-state clinical improvement in paired PD recordings
Influence diagnostic	M1 low-beta group × state	After removing one high-leverage observation: *χ*^2^(1) = 6.11, *p* = 0.014	Primary PSD result remained significant
Leave-one-out	M1 low-beta group × state	Significance retained in 38/38 iterations	Within-sample robustness of the primary PSD result
Clinical association	ΔMDS-UPDRS III vs. ΔM1 low-beta PSD	*β* = −0.21 per 1 dB; 95% CI [−1.37, 0.96]; *p* = 0.721	No significant association
Clinical association	ΔMDS-UPDRS III vs. Δ high-beta wPLI edge	*β* = −0.16 per 0.01 unit; 95% CI [−0.67, 0.35]; *p* = 0.523	No significant association

### EEG quality-control (QC) audit and segment-duration sensitivity analyses

The revision-stage quality-control (QC) audit verified three ON-state PD recordings shorter than the target 90 s, including two PIGD recordings and one TD recording. After excluding these three participants as complete paired subjects, the M1 low-beta group × state interaction remained directionally consistent and significant (*β* = −1.50, 95% CI [−2.74, −0.248], *p* = 0.021). In the uniform 60-s analysis, the M1 low-beta interaction was also directionally consistent and significant (*β* = −1.22, 95% CI [−2.20, −0.235], *p* = 0.017). SMA low-beta sensitivity results were similarly directionally consistent (original 90-s analysis: *β* = −1.65, *p* = 0.009; 90-s exclusion sensitivity: *β* = −1.72, *p* = 0.012; 60-s standardized analysis: *β* = −1.50, *p* = 0.005). The primary wPLI trait endpoint remained directionally consistent but non-significant across datasets (original 90-s analysis: *β* = −0.013, *p* = 0.186; 90-s exclusion sensitivity: *β* = −0.0145, *p* = 0.182; 60-s standardized analysis: *β* = −0.0136, *p* = 0.177). These sensitivity analyses strengthened the transparency of EEG data handling and supported the robustness of the primary M1 low-beta PSD finding, while the primary wPLI endpoint remained non-significant.

### Exploratory clinical associations

Across all PD participants with paired OFF/ON assessments, medication was associated with significant motor improvement, with a median OFF–ON change in MDS-UPDRS III of 9.0 points (interquartile range, 4.0–13.0; Wilcoxon signed-rank test, *p* < 0.001). However, exploratory regression analyses did not identify significant associations between electrophysiological change measures and clinical improvement. Specifically, ΔM1 low-beta PSD was not significantly associated with ΔMDS-UPDRS III (*β* = −0.21 points per 1 dB, 95% CI, −1.37 to 0.96; *p* = 0.721). Similarly, high-beta ΔwPLI between the right cerebellar Crus I and right lobule VI was not significantly associated with ΔMDS-UPDRS III (*β* = −0.16 points per 0.01 unit, 95% CI, −0.67 to 0.35; *p* = 0.523), as was shown in Table 3. Supplementary Figure S1 illustrates substantial inter-individual variability, with fitted slopes close to zero and confidence intervals crossing zero.

## Discussion

The principal finding of this study is a subtype-related difference in medication-state modulation of M1 low-beta PSD within a standardized OFF/ON design. This result was robust to excluding the three participants with shorter ON-state recordings and to a uniform 60-s re-analysis. The finding should be interpreted as a candidate electrophysiological correlate of subtype-related medication-state modulation, not as evidence for mutually exclusive circuits or a marker for clinical diagnosis.

This interpretation is consistent with the broader view that tremor, dopamine responsiveness, DBS effects, and beta-band physiology are not mutually exclusive across PD motor subtypes. Beta activity is relevant to bradykinesia, rigidity, motor state, and dopaminergic modulation, but it is not specific to akinetic-rigid symptoms or to the PIGD phenotype alone. The supportive SMA low-beta result points to a broader motor-cortical pattern, but it remains secondary to the prespecified M1 endpoint.

The connectivity findings require greater caution. The primary ipsilateral cerebellum-M1 low-beta wPLI trait endpoint remained non-significant, and NBS did not identify a family-wise-error-corrected network-level component. Exploratory edge-wise wPLI effects were sparse and edge-specific. These observations may help generate hypotheses about cerebellum-related network modulation, but they do not establish a definitive cerebellar mechanism.

This cautious interpretation is further reinforced by the methodological constraints inherent to cerebellar source-space EEG. In the present study, source imaging was performed without individual MRI, using the ICBM152 template and a three-shell spherical head model as a compromise between reproducibility and spatial precision. Template registration error, the simplified head model, and residual source leakage may all affect the absolute values and spatial specificity of cerebellum-related connectivity estimates. Although wPLI was selected to reduce spurious zero-lag coupling, it cannot fully eliminate uncertainty in source attribution or ROI mixing. The mechanistic implications of the exploratory cerebellar connectivity findings should therefore remain provisional.

From a clinical perspective, the present findings provide candidate physiological signals and methodological guidance for larger, MRI-informed, preregistered EEG studies of PD motor subtypes. The negative clinical association analyses argue against interpreting the current EEG measures as stand-alone indicators of treatment response in this cohort. Future studies with larger samples, individualized anatomy, balanced medication-state ordering when feasible, and independent replication will be needed before clinical translation can be considered.

Several limitations should be acknowledged. First, the sample size was modest, and the OFF-to-ON recording sequence was fixed. Second, three ON-state recordings were shorter than the target 90-s duration after preprocessing; sensitivity analyses excluding the affected paired subjects and using uniform 60-s data did not materially change the primary M1 low-beta finding. Third, exact upstream bad-channel and ICA-removal histories were not fully recoverable from the saved post-preprocessing files unless explicitly stored in metadata. Fourth, 64-channel EEG, template MRI, and a spherical head model limit spatial precision, especially for cerebellar source localization; no individual MRI was available. Fifth, the analysis was a prespecified source-space ROI analysis and did not include scalp-level 64-channel mass-univariate screening. Sixth, mixed/unknown subtype participants were not included in PIGD-vs-TD inferential models. Finally, clinical association analyses were negative, so the present findings cannot establish a stand-alone indicator of treatment response.

## Conclusion

In this source-space resting-state EEG study, TD and PIGD Parkinson’s disease showed distinct medication-state modulation of motor-cortical low-beta power under a fixed OFF/ON protocol. The prespecified M1 low-beta endpoint emerged as the clearest and most internally consistent subtype-related electrophysiological signal in this cohort. By contrast, cerebellum-related connectivity findings remained exploratory and were not supported at the network level. These results support further validation of motor-cortical low-beta modulation as a candidate electrophysiological correlate of subtype-related medication-state modulation in larger and methodologically more rigorous cohorts.

## Data Availability

The de-identified data and main analysis scripts supporting the conclusions of this article are not publicly available because of ethical and privacy restrictions. Requests to access the datasets and scripts should be directed to the corresponding author, Ganqin Du, Dugq99@haust.edu.cn.
