# Supplementary material for: Distinct medication-state modulation of motor-cortical low-beta power in tremor-dominant and postural instability/gait difficulty Parkinson’s disease: a source-space resting-state EEG study
Source: Front Neurol. 2026 Jun 25;17:1841473. doi: 10.3389/fneur.2026.1841473 (PMC13345854; doi:10.3389/fneur.2026.1841473)

**Supplementary Material**

**Supplementary Methods: EEG quality-control audit and segment-duration sensitivity analyses**

We performed a revision-stage audit of saved post-preprocessing EEG files and metadata to document recording availability, final saved segment duration, sampling rate, channels retained, and recoverable ICA metadata. The audit was read-only and did not modify source data. Segment-duration sensitivity analyses included a 90-s analysis excluding the affected paired participants and a uniform 60-s analysis. Inferential models were restricted to PIGD and TD participants with complete OFF/ON pairs; HC and mixed or unknown subtypes were not included in PIGD-vs-TD inferential models.

PSD sensitivity models used participant-specific random intercepts with outcome ~ group × state + age + sex + disease duration + (1|subject_id), using PIGD and OFF as reference levels. The primary wPLI trait model used outcome ~ group + age + sex + disease duration. Edge-wise wPLI analyses were exploratory and used within-band BH-FDR correction where the edge family was reconstructable.

**Supplementary Results: EEG QC and sensitivity analyses**

The QC audit identified three ON-state PD recordings shorter than the target 90 s, including two PIGD recordings and one TD recording. Excluding these paired participants and using a uniform 60-s dataset did not materially change the primary M1 low-beta PSD finding. The primary wPLI trait endpoint remained nonsignificant across analyses.

**Supplementary Table S1. EEG data availability and post-preprocessing quality-control summary**

| **Stratum** | **Recordings** | **Readable files** | **>=90 s data** | **Shorter retained** | **Duration, s** | **Sampling rate, Hz** | **Channels retained** | **Current ICA components** |
| --- | --- | --- | --- | --- | --- | --- | --- | --- |
| PIGD OFF | 20 | 20/20 | 20/20 | 0 | 179.99 (139.34-182.1); range 90.37-299.96 | 500 | 64 (64-64); range 64-64 | 62 (60-63); range 56-64 |
| PIGD ON | 20 | 20/20 | 18/20 | 2 | 179.38 (97.39-181.07); range 86.33-300.56 | 500 | 64 (64-64); range 64-64 | 59 (58-60); range 50-64 |
| TD OFF | 18 | 18/18 | 18/18 | 0 | 180.03 (165.88-190.33); range 92.71-309.42 | 500 | 64 (64-64); range 64-64 | 62 (60-63); range 55-64 |
| TD ON | 18 | 18/18 | 17/18 | 1 | 178.51 (124.09-185.89); range 60.19-310.5 | 500 | 64 (64-64); range 64-64 | 62 (56-63); range 52-64 |
| HC | 12 | 12/12 | 12/12 | 0 | 314.18 (292.49-316.24); range 184.64-486.46 | 500 | 64 (64-64); range 64-64 | 62 (60-62); range 53-63 |

Note. Values summarize the saved post-preprocessing recordings available for analysis. Exact upstream bad-channel deletion counts and removed ICA-component counts were not recoverable from the saved post-preprocessing files unless explicitly stored in metadata; these unavailable fields were not inferred or coded as zero. Anonymous audit identifiers for the three shorter ON-state PD recordings were AR-02, AR-20, and TD-13; no personal names are reported.

**Supplementary Table S2. Segment-duration sensitivity analyses for key PSD and wPLI endpoints**

| **Endpoint** | **Dataset** | **Subjects** | **Recordings** | **β** | **95% CI** | **p** | **Direction** | **Interpretation** |
| --- | --- | --- | --- | --- | --- | --- | --- | --- |
| M1 low-beta relative PSD | original_90s_all | 38 | 76 | -1.45 | 95% CI [-2.61, -0.299] | p = 0.016 | negative | direction consistent with original 90s |
| M1 low-beta relative PSD | 90s_excluding_short_segments | 35 | 70 | -1.5 | 95% CI [-2.74, -0.248] | p = 0.021 | negative | direction consistent with original 90s |
| M1 low-beta relative PSD | 60s_standardized | 38 | 76 | -1.22 | 95% CI [-2.2, -0.235] | p = 0.017 | negative | direction consistent with original 90s |
| SMA low-beta relative PSD | original_90s_all | 38 | 76 | -1.65 | 95% CI [-2.87, -0.443] | p = 0.009 | negative | direction consistent with original 90s |
| SMA low-beta relative PSD | 90s_excluding_short_segments | 35 | 70 | -1.72 | 95% CI [-3.01, -0.42] | p = 0.012 | negative | direction consistent with original 90s |
| SMA low-beta relative PSD | 60s_standardized | 38 | 76 | -1.5 | 95% CI [-2.53, -0.477] | p = 0.005 | negative | direction consistent with original 90s |
| Ipsilateral cerebellum-M1 low-beta wPLI | original_90s_all | 38 | 38 | -0.013 | 95% CI [-0.0327, 0.00661] | p = 0.186 | negative | direction consistent with original 90s |
| Ipsilateral cerebellum-M1 low-beta wPLI | 90s_excluding_short_segments | 35 | 35 | -0.0145 | 95% CI [-0.0361, 0.00713] | p = 0.182 | negative | direction consistent with original 90s |
| Ipsilateral cerebellum-M1 low-beta wPLI | 60s_standardized | 38 | 38 | -0.0136 | 95% CI [-0.0337, 0.00648] | p = 0.177 | negative | direction consistent with original 90s |

Note. Primary endpoint and primary wPLI trait rows are presented without q values because they were prespecified model tests rather than exploratory edge-wise families. The original main-text p value for the prespecified wPLI endpoint was based on the likelihood-ratio test, whereas the sensitivity-table p values are coefficient-level p values from comparable covariate-adjusted trait models.

**Supplementary Table S3. Exploratory edge-wise wPLI sensitivity results**

Full exploratory edge-wise sensitivity results are provided in Supplementary Table S3 as a separate Excel file. q values are used only for exploratory edge-wise within-band BH-FDR correction.

**Supplementary Figure Legends**

Supplementary Figure S1. Exploratory associations between electrophysiological change and motor improvement. Clinical change in MDS-UPDRS III was defined as OFF-ON, such that larger positive values indicate greater motor improvement. Exploratory regression analyses did not identify significant associations between electrophysiological change measures and clinical improvement.


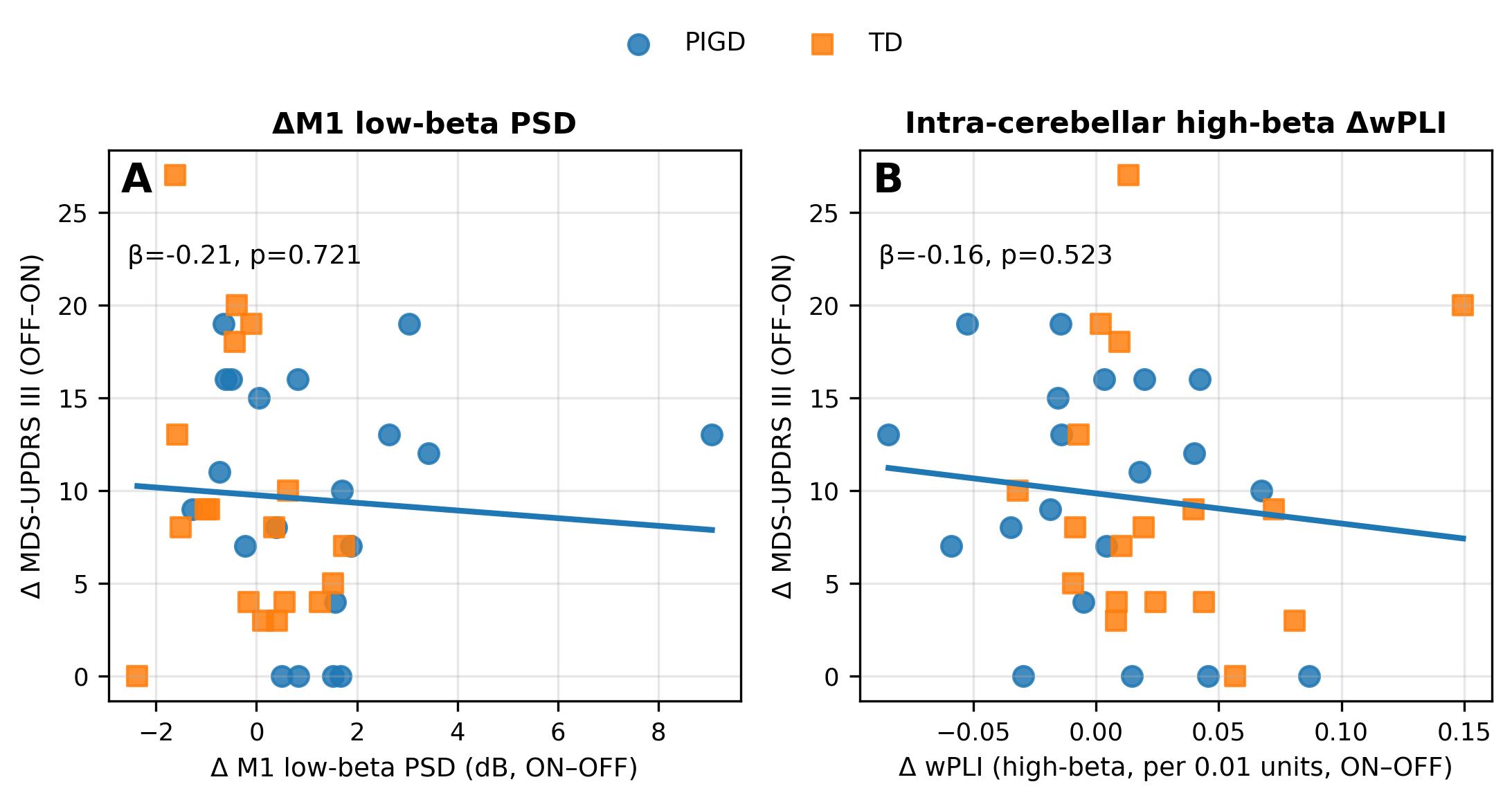

Supplement: Supplementary file 1 [file Table_1.DOCX]
